# Octachlorinated Metal Phthalocyanines (M = Co, Zn, VO): Crystal Structures, Thin-Film Properties, and Chemiresistive Sensing of Ammonia and Hydrogen Sulfide

**DOI:** 10.3390/s26010008

**Published:** 2025-12-19

**Authors:** Tatiana Kamdina, Darya Klyamer, Aleksandr Sukhikh, Pavel Popovetskiy, Pavel Krasnov, Tamara Basova

**Affiliations:** 1 Nikolaev Institute of Inorganic Chemistry, Siberian Branch of Russian Academy of Sciences, 3, Acad. Lavrentiev Ave., 630090 Novosibirsk, Russia; kamdinatana@gmail.com (T.K.); klyamer@niic.nsc.ru (D.K.); a_sukhikh@niic.nsc.ru (A.S.); popovetskiy@niic.nsc.ru (P.P.); 2 Faculty of Natural Sciences, Novosibirsk State University, 1, Pirogova Str., 630090 Novosibirsk, Russia; 3 International Research Center of Spectroscopy and Quantum Chemistry, Siberian Federal University, 26 Kirensky St., 660074 Krasnoyarsk, Russia; kpo1980@gmail.com

**Keywords:** octachlorinated phthalocyanines, single crystal structure, thin films, chemiresistive sensors, ammonia, hydrogen sulfide

## Abstract

Octachlorinated metal phthalocyanines (MPcCl_8_, M = Co, Zn, VO) represent an underexplored class of functional materials with promising potential for chemiresistive sensing applications. This work is the first to determine the structure of single crystals of CoPcCl_8_, revealing a triclinic (P-1) packing motif with cofacial molecular stacks and an interplanar distance of 3.381 Å. Powder XRD, vibrational spectroscopy, and elemental analysis confirm phase purity and isostructurality between CoPcCl_8_ and ZnPcCl_8_, while VOPcCl_8_ adopts a tetragonal arrangement similar to its tetrachlorinated analogue. Thin films were fabricated via physical vapor deposition (PVD) and spin-coating (SC), with SC yielding highly crystalline films and PVD resulting in poorly crystalline or amorphous layers. Electrical measurements demonstrate that SC films exhibit n-type semiconducting behavior with conductivities 2–3 orders of magnitude higher than PVD films. Density functional theory (DFT) calculations corroborate the experimental findings, predicting band gaps of 1.19 eV (Co), 1.11 eV (Zn), and 0.78 eV (VO), with Fermi levels positioned near the conduction band, which is consistent with n-type character. Chemiresistive sensing tests reveal that SC-deposited MPcCl_8_ films respond reversibly and selectively to ammonia (NH_3_) and hydrogen sulfide (H_2_S) at room temperature. ZnPcCl_8_ shows the highest NH_3_ response (45.3% to 10 ppm), while CoPcCl_8_ exhibits superior sensitivity to H_2_S (LOD = 0.3 ppm). These results suggest that the films of octachlorinated phthalocyanines produced by the SC method are highly sensitive materials for gas sensors designed to detect toxic and corrosive gases.

## 1. Introduction

Halogenated phthalocyanine-based materials have shown great potential for use in a variety of applications, including organic field-effect transistors, sensors, and biologically related applications [1,2,3]. The development of new materials based on MPcHal_x_ is always a focus, as it allows for further improvement of the performance characteristics and expansion of the application areas of these materials. Their inherent chemical and thermal stability, combined with tunable electronic properties, also makes them valuable building blocks for covalent organic frameworks [4,5,6,7].

Halogenation is known to enhance oxidative stability by shifting oxidation potentials to the anodic region [8] and significantly improves sensitivity toward reducing gases such as ammonia and hydrogen sulfide [2,9]. In recent years, fluoro-substituted metal phthalocyanines (MPcF_x_, x = 4, 8, 16) have attracted considerable research interest, with numerous studies reporting on their structural [10,11], thermal [12,13], optical [14], and electrical properties [15], as well as their application in thin-film electronic devices [16]. In contrast, investigations into chloro-substituted MPcCl_x_ have largely focused on synthetic methodologies and optical characteristics, while systematic studies of their crystal structures and thin-film morphology, particularly in technologically relevant thickness ranges, remain comparatively scarce. This disparity is partly attributable to the historical use of chlorinated phthalocyanines as green organic pigments in industrial applications [17,18,19].

Nevertheless, chlorinated MPcs exhibit promising functional properties beyond pigmentation. For instance, chlorinated bis(phthalocyaninates) of rare-earth metals display strong absorption in the 680–700 nm range within the so-called “therapeutic window” for photodynamic therapy and demonstrate enhanced superoxide anion generation compared to their non-halogenated analogues [20,21]. Moreover, certain mixed halogen derivatives, such as MCl_2_PcF_16_, MCl_2_PcCl_16_, and MF_2_PcCl_16_ (M = Sn, Ge), have been shown to possess optical limiting capabilities [22]. Nonlinear optical behavior has also been reported for lanthanide-based octachlorinated phthalocyanines [23].

Research on thin films of chlorinated phthalocyanine has primarily addressed ultrathin layers (typically <5 nm), emphasizing interfacial interactions with substrates and surface phenomena [24,25,26]. However, there is a notable gap in understanding the structural and morphological characteristics of thicker films (50–200 nm), despite their widespread use as active layers in organic field-effect transistors (OFETs) and chemiresistive sensors. Notably, tetrachloro-substituted ZnPcCl_4_ has demonstrated superior sensitivity toward ammonia compared to both unsubstituted and fluorinated analogues, highlighting the potential of chlorinated MPcs in chemical sensing [2].

Our previous work has contributed to this field through structural investigations of single crystals and thin films of tetrachloro-substituted phthalocyanines with Cl-substituents in peripheral (MPcCl_4_-p, M = Zn, VO) and non-peripheral (MPcCl_4_-np, M = Zn, VO) [2,27]. Additionally, VOPcCl_16_ films have been evaluated as active components in OFET architectures [28]. At the same time, structure and properties of octachloro-substituted phthalocyanines have not been extensively studied, even though synthetic protocols for Cu(II), Co(II), Ni(II), and Zn(II) octachlorophthalocyanines have been well established [29]. To date, only the Lyubovskaya group has reported a structure of crystalline anionic salt of an octachlorinated derivative: {cryptand(Na^+^)}_2_[Sn^IV^Cl_2_(TPyzPzCl_8_^4−^)]^2−^·2C_6_H_4_Cl_2_ (triclinic, P1^−^) [30].

Conductivity studies of chloro substituted cobalt phthalocyanines were provided by Achar and co-workers [31]. It was found that CoPcCl_8_ exhibits a linear variation in electrical conductivity in the temperature range 30–200 °C. The room temperature electrical conductivities observed for CoPcCl_4_, CoPcCl_8_ and CoPcCl_16_ are 10^4^, 10^2^ and 10^6^ times higher than CoPc complex (CoPcCl_16_ ˃ CoPcCl_4_ ˃ CoPcCl_8_ ˃ CoPc). The sensor properties of octachloro-substituted metal phthalocyanines have also been practically unstudied. Bouvet and his group of researchers prepared bilayer configuration organic heterojunctions based on octachlorinated metallophthalocyanines (MPcCl_8_; M = Co, Cu and Zn) as a sublayer and lutetium bis-phthalocyanine (LuPc_2_) as a top layer [32]. While the metal has little effect on baseline device performance, it dictates the NH_3_ sensing mechanism: CoPcCl_8_ enables n-type response to NH_3_, CuPcCl_8_ shows p-type response, ZnPcCl_8_ exhibits ambipolar behavior. In our recent work we investigated the effect of the central metal ion on crystal features and conductivity for MPcF_8_ (M = Co, Zn, VO) [33]. The lateral DC conductivity of ZnPcF_8_ and VOPcF_8_ films was similar, whereas the conductivity of CoPcF_8_ films was significantly higher.

Thus, an analysis of the literature has shown that films of octachloro-substituted phthalocyanines and especially structure-property-sensor performance correlation are insufficiently studied in comparison with fluoro-substituted analogues. Their crystallographic structure, film morphology, and chemiresistive properties remain virtually unexplored, despite expectations that eight chlorine atoms strongly withdrawing electrons can cause profound changes in the electronic structure and intermolecular interactions compared with tetrachlorinated or fluorinated analogues. Unlike MPcF_8_ derivatives, where the high electronegativity and small size of fluorine favor planar, densely packed structures, the larger van der Waals radius and polarizability of chlorine in MPcCl_8_ can contribute to a different type of packing and, as a result, charge transfer. Moreover, the combined inductive effect of the eight chlorines is expected to significantly stabilize the boundary orbitals, potentially narrowing the band gap and shifting the Fermi level towards the conduction band, thereby providing n-type semiconductor properties that are rare among conventional phthalocyanines.

**Figure 1:**
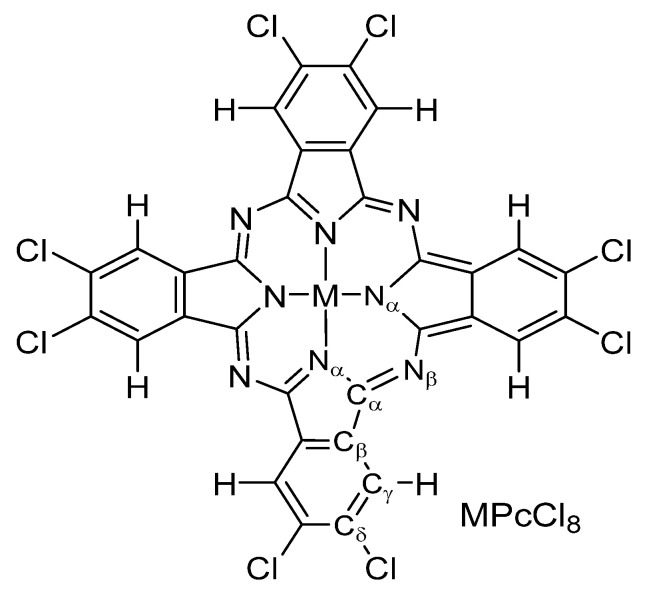
Structure of MPcCl_8_ (M = Co, Zn, VO).

In the present study, we address these critical gaps by reporting the synthesis of octachloro-substituted metal phthalocyanines MPcCl_8_ (M = Co, Zn, VO; Figure 1) and, for the first time, the single-crystal X-ray structure of CoPcCl_8_. All compounds were thoroughly characterized by elemental analysis, vibrational spectroscopy (IR and Raman), electronic absorption spectroscopy, and X-ray diffraction to confirm chemical composition and purity. Thin films were fabricated via both vacuum thermal evaporation and spin-coating techniques. The physicochemical properties of the bulk materials and their corresponding thin films were systematically investigated. Finally, the sensing performance of these films was evaluated as active layers in chemiresistive sensors for the detection of ammonia (NH_3_) and hydrogen sulfide (H_2_S).

## 2. Materials and Methods

### 2.1. Synthesis of Octachloro-Substituted Metal Phthalocyanines

MPcCl_8_ (M = Co, Zn, VO) were synthesized via a solution-based cyclotetramerization method. In a typical procedure, 4,5-dichlorophthalonitrile (Sigma-Aldrich, St. Louis, MO, USA, CAS 139152-08-2, ≥99%) and the corresponding metal chloride (CoCl_2_, ZnCl_2_, or VOCl_3_ Sigma-Aldrich, St. Louis, MO, USA) were refluxed in 1-chloronaphthalene (Sigma-Aldrich, St. Louis, MO, USA, CAS 90-13-1, ≥85 %) under constant stirring for 2 h. The crude products were purified using a Soxhlet extractor with sequential extraction in diethyl ether, acetone, and ethanol (24 h) (solvents were purchased from AO REAKHIM, Moscow, Russia) to remove unreacted precursors and by-products. The resulting complex was then heated under vacuum (10^−5^ Torr) at 300 °C. Final yields ranged from 55% to 70%. All compounds were characterized by elemental analysis, vibrational spectroscopy (IR and Raman), and X-ray diffraction to confirm chemical composition and purity. Elemental analysis results:

CoPcCl_8_ yield 71.3%. Elemental Anal. Calcd. for C_32_H_8_N_8_Cl_8_Co: C, 45.4; N, 13.2; H, 1.0. Found: C, 45.5; N, 12.9; H, 1.3.

ZnPcCl_8_ yield 69.5%. Elemental Anal. Calcd. for C_32_H_8_N_8_Cl_8_Zn: C, 45.0; N, 13.1; H, 1.0. Found: C, 45.1; N, 13.3; H, 1.3.

VOPcCl_8_ yield 54.7%. Elemental Anal. Calcd. for C_32_H_8_N_8_Cl_8_VO: C, 43.9; N, 12.8; H, 1.0. Found: C, 44.0; N, 12.4; H, 1.2.

Elemental analyses (C, H, N) were performed using a Thermo Finnigan Flash 1112 analyzer (Thermo Finnigan Italia S.p.A. Milan, Italy). IR spectra were recorded on a Bruker Vertex 80 FT-IR spectrometer (Bruker, Ettlingen, Germany) in transmission mode (KBr pellets).

### 2.2. Thin Film Fabrication

Thin films of MPcCl_8_ were prepared using two complementary techniques: spin-coating (SC) and physical vapor deposition (PVD). Films were deposited onto glass substrates (Deltalab, Barcelona, Spain, 18 × 18 mm) and glass substrates with interdigitated Pt electrodes (Metrohm DropSens, Oviedo, Asturias, Spain). Prior to deposition, the substrates were cleaned sequentially with sulfuric acid, distilled water and acetone, then boiled in isopropanol for 20 min, and finally dried at ambient temperature.

*Spin-coating:* A solution of 3.0 mg of MPcCl_8_ in 400 µL of tetrahydrofuran (THF, AO REAKHIM, Moscow, Russia) was deposited dropwise onto cleaned substrates using a microdispenser (Thermo Scientific, Saint-Petersburg, Russia). Films were formed by spinning at 2000 rpm for 60 s using an Elmi CM-6M centrifuge (SIA «ELMI», Riga, Latvia).

*Physical vapor deposition:* Films were thermally evaporated under high vacuum (10^−5^ Torr) using a VUP-5M deposition system. The MPcCl_8_ powders were evaporated at 450 °C for 2 h, resulting in uniform films with nominal thicknesses of about 50–80 nm.

### 2.3. Structural and Morphological Characterization

Single-crystal X-ray diffraction (SC-XRD) of CoPcCl_8_ was performed on a Bruker D8 Venture diffractometer (Billerica, MA, USA) (equipped with an Incoatec IµS 3.0 microfocus CuKα X-ray source (λ = 1.54178 Å) and a PHOTON III CPAD detector mounted on a three-circle kappa goniometer. A needle-shaped crystal (70 × 5 µm) was selected from the as-synthesized product. Data collection strategy consisted of several standard ω-scans with 0.5° wide frames. Temperature of single crystal sample was kept at 150 K during the experiment by an Oxford Cryosystems Cryostream 800 plus open-flow nitrogen cooler (Oxford Cryosystems, Oxford, UK). Data processing, including unit cell determination, integration, and multi-scan absorption correction, was performed using APEX3 v.2019.1-0 software package (Madison, WI, USA) [34]. Obtained *hklF* dataset was processed in Olex2 v.1.5 [35], with SHELXT v.2018/2 [36] and SHELXL v.2018/3 [37] used for structure solution and refinement, respectively. All non-hydrogen atoms were refined anisotropically, but ISOR restraints had to be placed on all carbon and nitrogen atoms due to the poor quality of the diffraction data. Hydrogen atoms were placed in idealized positions and refined using the “riding” model. Final CIF was deposited to the Cambridge Crystallographic Data Centre (CCDC) under No. 2503921 and can be downloaded for free at www.ccdc.cam.ac.uk/structures (accessed on 18 November 2025).

Powder X-ray diffraction (PXRD) patterns of polycrystalline samples and thin films were collected on a Bruker D8 Advance diffractometer (Billerica, MA, USA) (CuKα radiation, λ = 1.5406 Å) equipped with a LynxEye XE-T silicon strip detector. Powder samples were gently ground in an agate mortar with a minimal amount of ethanol and spread as a thin layer on flat surface of a standard sample holder. Diffraction data were recorded over a 2θ range of 3–40° with a step size of 0.02°.

The topology of MPcCl_8_ films was studied in the semi-contact mode using the NTEGRA Prima II atomic force microscope (NT-MDT, Moscow, Russia). The MFM-01 probe was used with the following parameters: probe length—225 μm, width—28 mm, thickness—3 μm, force constant—1–5 N/m, and resonant frequency—47–90 kHz.

### 2.4. Quantum-Chemical Calculations

Quantum-chemical computations of the band structure for MPcCl_8_ crystals (where M = Co, Zn, and VO) were carried out using the OpenMX package (version 3.9) based on density functional theory (DFT) [38], with the PBE functional employed for the exchange-correlation energy [39]. The calculations utilized norm-conserving pseudopotentials [40,41,42,43,44,45] and an optimized pseudo-atomic orbital basis set labeled as “standard” in OpenMX [46,47]. Due to the closed-shell configuration of zinc, spin-unpolarized calculations were performed for ZnPcCl_8_. In contrast, spin-polarized DFT+*U* calculations were applied to CoPcCl_8_ and VOPcCl_8_, following Dudarev’s approach [48,49]. A Hubbard *U* parameter of 5 eV was selected, consistent with values previously established in the literature for analogous phthalocyanine systems. In particular, comprehensive studies on Co-phthalocyanine (CoPc) have demonstrated that the *U*_eff_ value in the range of 4–6 eV provides optimal agreement with experimental valence-band photoelectron spectroscopy data and hybrid functional (e.g., B3LYP, HSE06) results for the position and ordering of Co 3d states, as well as structural and magnetic properties [50,51,52]. A value of *U*_eff_ = 6 eV was explicitly shown to best reproduce both the experimental bond lengths and the Co 3d spectral features in CoPc [50], while a systematic comparison across transition-metal phthalocyanines confirmed that the appropriate *U*_eff_ was system-dependent and typically falls within 4–6 eV for Co-containing systems [51]. Similarly, for vanadyl phthalocyanines, *U* ≈ 5 eV has been successfully used to capture the correct electronic structure and magnetic behavior [53]. Given the strong structural and electronic similarity between unsubstituted CoPc/VOPc and their octachlorinated derivatives, the choice of *U* = 5 eV (within ±1 eV of the values validated in prior work) is well-justified for reliably describing the correlated 3d electrons in CoPcCl_8_ and VOPcCl_8_. Additionally, dispersion effects were accounted for using the DFT-D3 correction [54,55]. Structural relaxation was performed until the residual forces on atoms fell below 3 × 10^−4^ a.u.

Furthermore, electrical transport properties were derived from the computed band structures. Special attention was given to the variation in the Seebeck coefficient with chemical potential. These estimates were made within the Boltzmann transport formalism [56] using the BoltzTraP code (version 1.2.5) [57].

## 3. Results and Discussion

### 3.1. Characterization of CoPcCl_8_, ZnPcCl_8_, and VOPcCl_8_

#### 3.1.1. Crystal Structure of CoPcCl_8_

**Figure 2:**
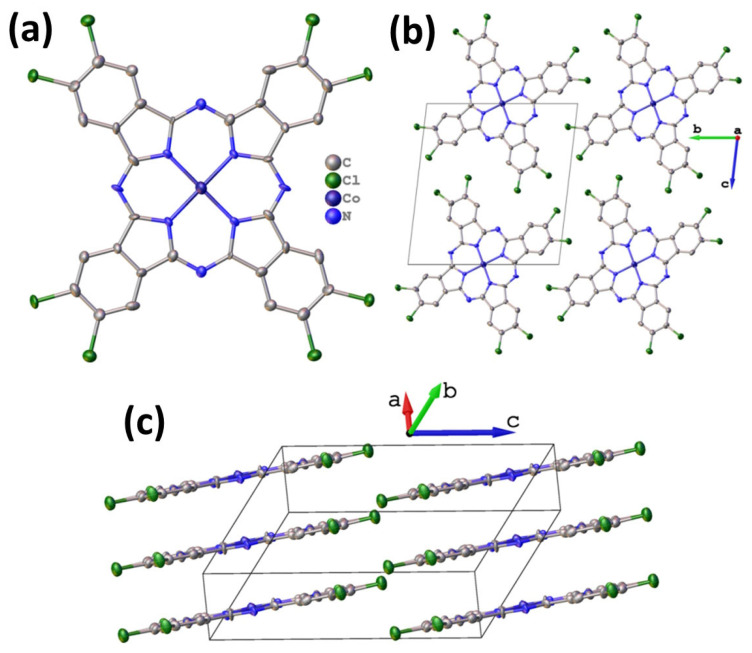
Molecular structure (**a**) and packing diagrams (**b**,**c**) for CoPcCl_8_.

Octachlorinated metal phthalocyanines exhibit significantly lower volatility compared to their fluoro-substituted analogues. Consequently, obtaining high-quality single crystals suitable for X-ray diffraction analysis is exceptionally challenging. Nevertheless, a small needle-shaped crystal of CoPcCl_8_ was fortuitously identified in the crude synthesis product, enabling the first single-crystal structure determination of an octachloro-substituted cobalt phthalocyanine. Despite the modest quality of the diffraction data attributable to the crystal’s small size and potential disorder the inherent rigidity of the phthalocyanine macrocycle allowed for a chemically reasonable structural model to be refined without imposing geometric restraints. Unit cell parameters and refinement details are summarized in Table 1. CoPcCl_8_ crystallizes in the P-1 space group with Z = 1. The structure is isostructural with its fluoro-substituted analogue CoPcF_8_ [33], confirming that halogen substitution at the peripheral positions does not alter the fundamental packing motif in this series. The molecules are packed in uniform stacks along the a axis (Figure 2) with an interplanar distance of 3.381 Å between adjacent macrocycles and a packing angle (defined as an angle between the packing direction and the normal to the molecule plane) of 24.18°.

#### 3.1.2. Powder XRD Analysis of CoPcCl_8_, ZnPcCl_8_, and VOPcCl_8_

**Figure 3:**
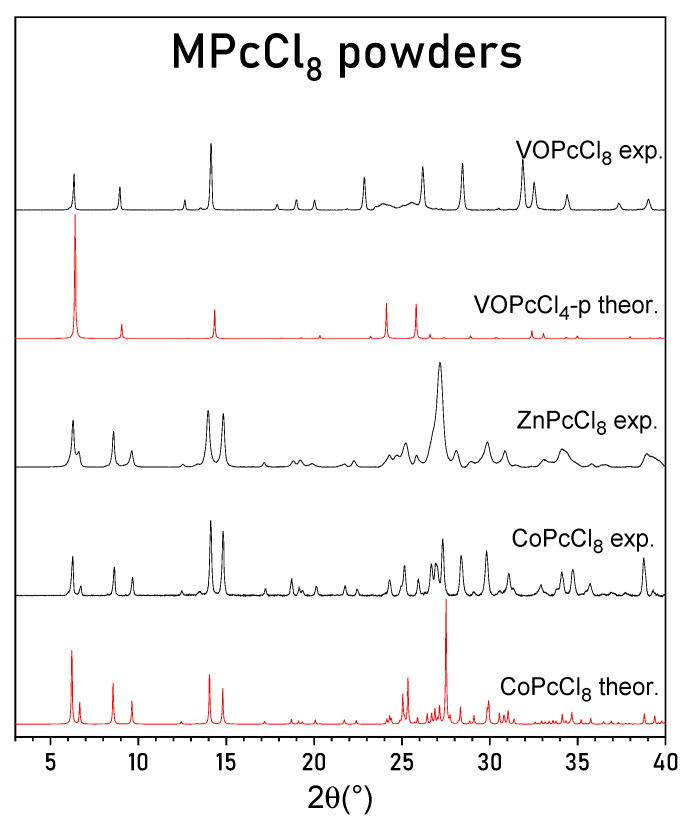
Experimental powder diffraction patterns for CoPcCl_8_, ZnPcCl_8_ and VOPcCl_8_ and calculated diffraction patterns for CoPcCl_8_ and VOPcCl_4_-p.

PXRD patterns of CoPcCl_8_, ZnPcCl_8_, and VOPcCl_8_ are presented in Figure 3, in comparison with theoretical patterns calculated from the single-crystal structure of CoPcCl_8_ and from the known structure of VOPcCl_4_-p [27]. The experimental PXRD pattern of CoPcCl_8_ shows good agreement with the pattern simulated from its single-crystal structure. No extraneous diffraction peaks are observed, confirming that the polycrystalline sample consists of a single crystalline phase without detectable impurities.

Although the crude product of ZnPcCl_8_ exhibited a similar morphology—dark violet, needle-like crystals with a metallic luster—the crystals were substantially smaller, precluding even unit cell determination by single-crystal methods. Nevertheless, the PXRD pattern of ZnPcCl_8_ closely resembles that of CoPcCl_8_, indicating that these compounds are isostructural. Leveraging the CoPcCl_8_ structural model, we successfully indexed the diffraction peaks of ZnPcCl_8_ and refined its unit cell parameters, which are reported in Table 2.

The PXRD pattern of VOPcCl_8_ closely matches the calculated pattern for tetragonal VOPcCl_4_-p, indicating that VOPcCl_8_ also adopts a tetragonal unit cell with molecules arranged in stacks along the c axis. It is worth noting that the peaks on the VOPcCl_8_ diffraction pattern are not uniform. Two peaks in the 23–26° 2θ region are significantly broader than the others. These two peaks correspond to the (011) and (121) crystallographic planes and are the only peaks with an l index not equal to zero observed on the diffraction pattern. Their broadening suggests the presence of stacking faults or orientational disorder along the c-axis. A similar phenomenon has been reported for VOPcCl_4_-p, where vanadyl phthalocyanine molecules stack in a head-to-tail fashion with random “up”/“down” orientations, leading to diffuse scattering and a highly disordered structural model. Using the VOPcCl_4_-p crystal structure data, we indexed the VOPcCl_8_ diffraction pattern and refined its tetragonal unit cell parameters (Table 2).

#### 3.1.3. IR Spectra of MPcCl_8_

**Figure 4:**
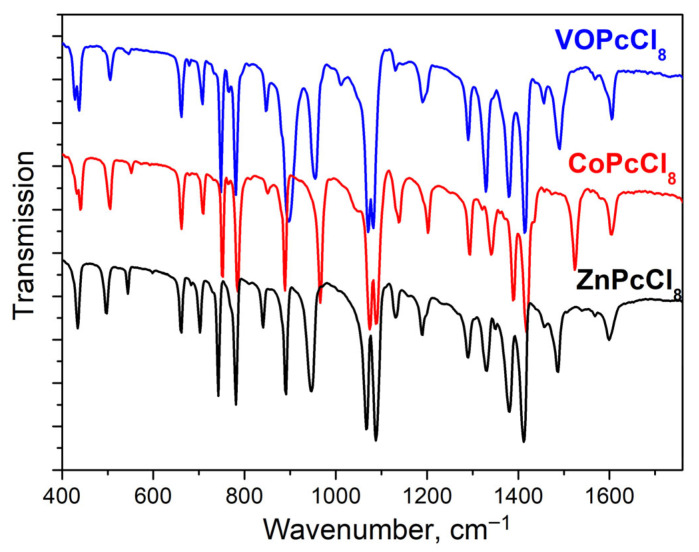
MPcCl_8_ IR-spectra in KBr pellets.

IR spectra of MPcCl_8_ were also studied (Figure 4). Band assignments supported by density functional theory (DFT) calculations are summarized in Table 3. Most vibrational modes originate from the phthalocyanine macrocycle and show minimal frequency shifts across the series (M = Co, Zn, VO). However, several key bands exhibit metal-dependent frequency variations. Like MPc and MPcF_x_ derivatives [58], the bands sensitive to the central metal are located in the spectral range from 1350 to 1550 cm^−1^, as they are associated with vibrations of atoms forming the inner cavity of the macrocycle. Apart from this, V = O vibrations is observed at 1012 cm^−1^.

### 3.2. Structural and Functional Characterization of CoPcCl_8_, ZnPcCl_8_, and VOPcCl_8_ Thin Films

Thin films of MPcCl_8_ (M = Co, Zn, or VO) were prepared using two distinct deposition techniques: physical vapor deposition (PVD) and solution-based spin-coating (SC) from dichloromethane. The structural properties of these films were investigated by X-ray diffraction (XRD), and their morphological and sensing characteristics were further analyzed using atomic force microscopy (AFM) and chemiresistive measurements.

#### 3.2.1. Structural Properties

**Figure 5:**
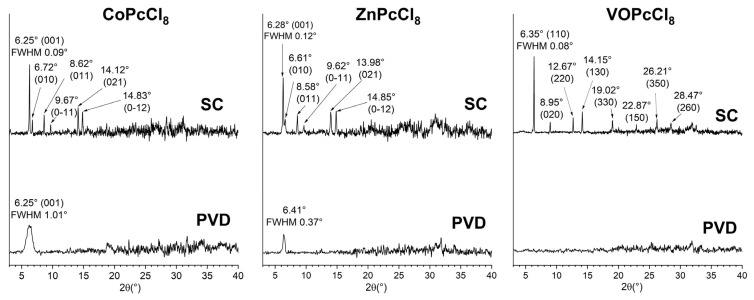
XRD patterns for PVD and SC of CoPcCl_8_, ZnPcCl_8_ and VOPcCl_8_ films.

XRD patterns of PVD-deposited CoPcCl_8_ and ZnPcCl_8_ exhibit a single broad diffraction peak, indicative of strong molecular orientation but poor crystallinity (Figure 5). In contrast, the PVD film of VOPcCl_8_ shows no discernible Bragg peaks, suggesting an amorphous structure. The peak position for CoPcCl_8_ (2θ = 6.25°) aligns closely with the (001) reflection calculated from single-crystal data, confirming that the PVD film retains the same crystalline phase as the bulk polycrystalline powder. Moreover, this alignment allows estimation of the molecular tilt angle relative to the substrate: CoPcCl8 molecules are oriented at approximately 76.8° with respect to the surface plane.

In contrast, the ZnPcCl_8_ PVD film displays a peak at 2θ = 6.41°, which deviates significantly from the expected position based on powder diffraction data. This shift implies the formation of another unknown crystal phase under PVD conditions. Spin-coated films of all three compounds, by comparison, display multiple sharp diffraction peaks that match well with reference powder patterns. This indicates that SC films adopt the same crystalline phase as the bulk powders, exhibit better crystallinity, and lack a preferred orientation. Using the Scherrer equation and accounting for instrumental broadening (0.05°), the coherent scattering region (CSR) sizes were estimated as follows: CoPcCl_8_: 8 nm (PVD) vs. 190 nm (SC); ZnPcCl_8_: 24 nm (PVD) vs. 110 nm (SC); VOPcCl_8_: 240 nm (SC only, as PVD film is amorphous). These results underscore the profound influence of deposition method on both crystallinity and phase formation.

**Figure 6:**
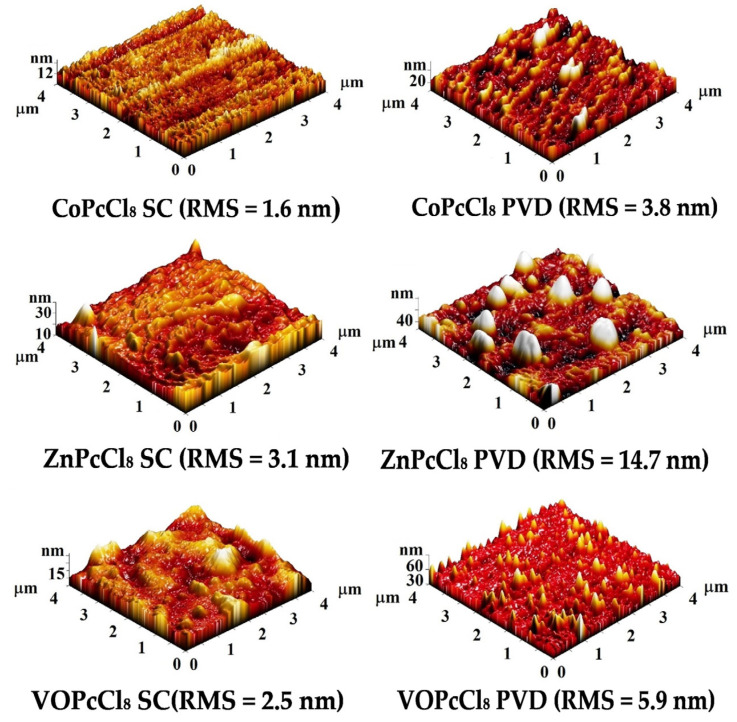
Three-dimensional AFM images of the surface of VOPcCl_8_, ZnPcCl_8_ and CoPcCl_8_ films.

Figure 6 shows AFM topography images of CoPcCl_8_, ZnPcCl_8_, and VOPcCl_8_ prepared by spin-coating (SC) and physical vapor deposition (PVD). SC films have a smoother surface (3D RMS roughness 1.6–3.1 nm) with a homogeneous layered morphology and densely packed crystallites. The CoPcCl_8_ SC film has the smoothest surface (1.6 nm), indicating excellent film uniformity. PVD films have a rougher surface (3D RMS roughness: 3.8–14.7 nm) showing island-like or columnar growth due to vapor-phase nucleation. The ZnPcCl_8_ PVD film has the roughest surface (14.7 nm) with large spherical inclusions, while the VOPcCl_8_ PVD film has sharp needle-like features probably due to the directional influence of the V=O group. The data indicate that both the central metal and the deposition method significantly influence the surface microrelief.

#### 3.2.2. Electrical and Sensor Properties

**Figure 7:**
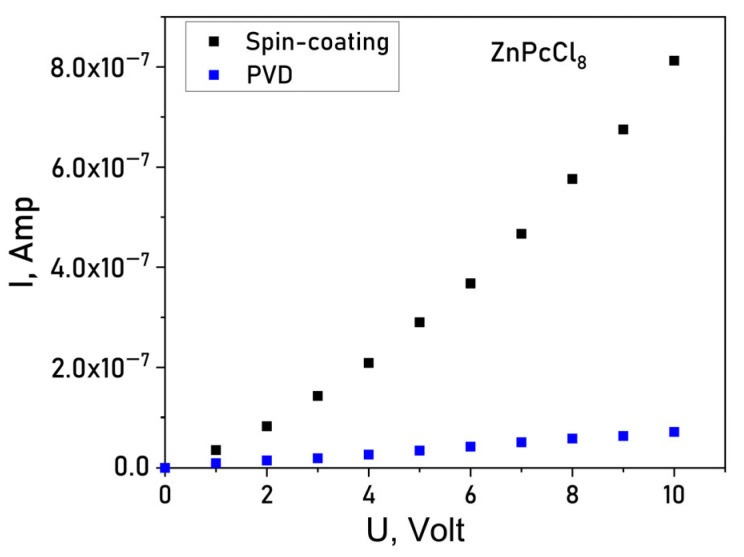
Current-voltage (I(V)) dependence for a ZnPcCl_8_ film.

To evaluate the impact of the central metal ion and deposition method on conductivity current-voltage (I–V) characteristics of the films deposited onto glass substrates with interdigitated Pt electrodes using Keithley 236 electrometer. I(V) dependences of a ZnPcCl_8_ film are shown in Figure 7 as an example. Spin-coated MPcCl_8_ films displayed lateral DC conductivities in the range of 1 × 10^−6^–5 × 10^−6^ Ω^−1^ m^−1^. In contrast, PVD-deposited films exhibit markedly lower conductivity (5 × 10^−9^–6 × 10^−9^ Ω^−1^ m^−1^). The conduction mechanism also differed between deposition methods: PVD films showed ohmic behavior over 0–10 V, whereas SC films followed a space-charge-limited (SCL) conduction model, consistent with their higher crystallinity and grain-boundary-mediated transport. Notably, PVD films demonstrated negligible chemiresistive response and were deemed unsuitable for sensor applications. Consequently, all subsequent gas-sensing studies focused on SC-deposited films.

**Figure 8:**
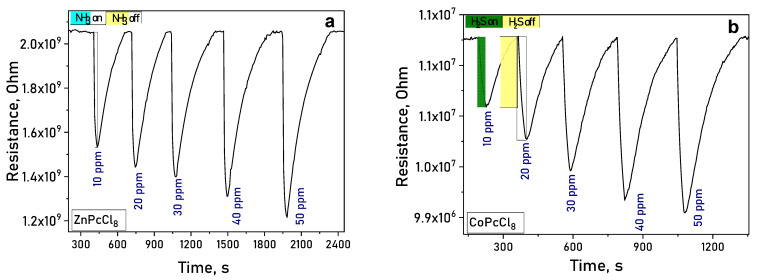
(**a**) Change in the resistance of ZnPcCl_8_ (**a**) and CoPcCl_8_ (**b**) films during the introduction of ammonia and subsequent air purging.

Upon exposure to ammonia (NH_3_) or hydrogen sulfide (H_2_S), the electrical resistance of MPcCl_8_ films decreased reversibly (Figure 8a,b), confirming n-type semiconducting behavior. In atmospheres of NH_3_ and H_2_S—both electron-donating gases—the resistance of n-type semiconductor films decreases, whereas the resistance of p-type semiconductors increases [59].

Previous studies have shown that peripherally substituted cobalt phthalocyanine (CoPcCl_4_-p) exhibits n-type behavior, while its non-peripherally substituted analog (CoPcCl_4_-np) behaves as a p-type semiconductor [60]. Furthermore, it has been demonstrated that fluorinated phthalocyanines (MPcF_x_) can transfer from p-type to ambipolar and ultimately to n-type semiconductors depending on the degree of fluorination [61,62,63]. Chlorosubstituted phthalocyanines have been less extensively investigated. One notable exception is the work of Pakhomov et al. [64], who observed a decrease in the electrical conductivity of CuPcCl_16_ films upon exposure to NO_2_. Since NO_2_ is an electron-withdrawing gas, this decrease in conductivity implies that the majority charge carriers in CuPcCl_16_ are electrons—indicating n-type behavior—unlike unsubstituted CuPc or peripherally tetrachlorinated CuPcCl_4_-p, which typically exhibit p-type characteristics. Importantly, semiconducting behavior depends not only on the number and position of halogen substituents but also on the nature of the central metal ion. For instance, whereas CoPcCl_4_-p behaves as an n-type semiconductor, its zinc analog ZnPcCl_4_-p exhibits p-type behavior [2]. Additionally, in heterojunction gas sensors using 2,3,9,10,16,17,23,24-octachloro-substituted phthalocyanines as bottom layers, cobalt and copper derivatives showed opposite responses to ammonia [65]: the cobalt complex acted as a p-type material, while the copper analog displayed n-type characteristics. We provide quantum-chemical calculations in attempt to suggest semiconductor behavior of MPcCl_8_.

#### 3.2.3. DFT Calculation of the Band Structure of CoPcCl_8_, ZnPcCl_8_, and VOPcCl_8_ Crystals

**Figure 9:**
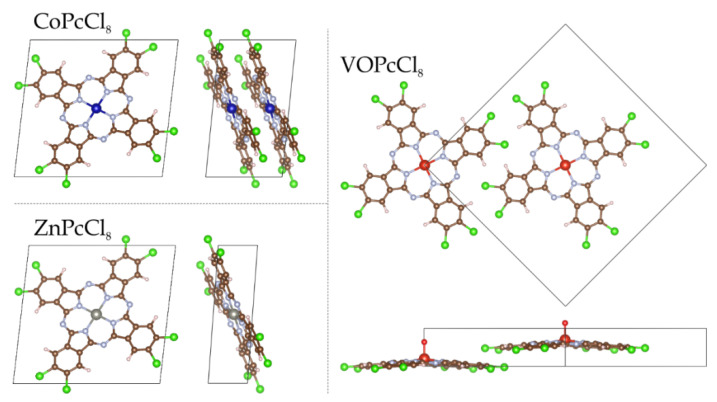
Top and side views of the geometric structures of MPcCl_8_ (M = Co, Zn, VO), showing the molecular plane from above and the lateral arrangement of the peripheral atoms from a side perspective.

X-ray diffraction data were employed to generate computational models of the MPcCl_8_ crystals (M = Co, Zn, VO). Initially, the atomic coordinates within each system were relaxed under fixed lattice parameters: for ZnPcCl_8_ and VOPcCl_8_, optimizations were performed within the unit cell, whereas for CoPcCl_8_—a system requiring explicit treatment of spin correlations—a 2 × 1 × 1 supercell was utilized (Figure 9). These calculations were carried out using a Monkhorst-Pack *k*-point grid of 5 × 5 × 5 to adequately sample the first Brillouin zone [66].

The crystal structure of CoPcCl_8_ was modeled using a 2 × 1 × 1 supercell to properly account for the collective spin state of the system. The unit cell of CoPcCl_8_ contains a single phthalocyanine molecule; a spin-polarized calculation on this unit cell inherently yields a high-spin state (*S* = 1/2), arising from the unpaired electron localized on the cobalt center. In the 2 × 1 × 1 supercell, which encompasses two molecules, two distinct total spin states become accessible: a singlet state (*S* = 0) with antiparallel alignment of the Co-centered spins, and a triplet state (*S* = 1) with parallel alignment. Preliminary calculations revealed that the *S* = 0 state is energetically favored. Consequently, all subsequent calculations for CoPcCl_8_ were performed in the spin-singlet configuration.

A similar analysis was carried out for VOPcCl_8_. In contrast to CoPcCl_8_, the unit cell of VOPcCl_8_ already contains two molecules, enabling direct assessment of both parallel and antiparallel spin configurations without the need for a supercell. Preliminary computations confirmed that, likewise, the *S* = 0 state is more stable than the *S* = 1 state, due to strong intermolecular exchange interactions between the vanadium centers. Accordingly, all subsequent calculations for VOPcCl_8_ were also conducted in the spin-singlet state.

**Figure 10:**
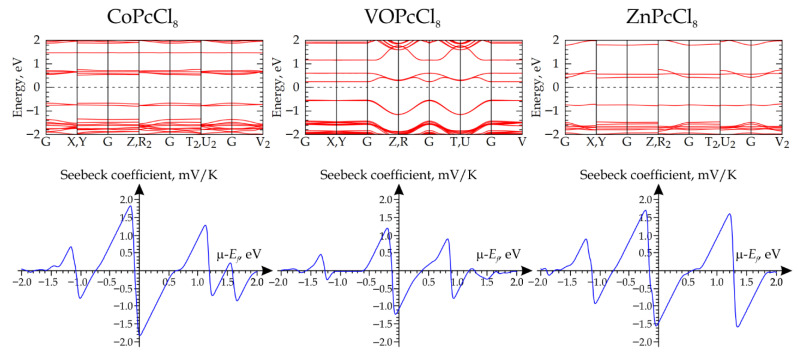
Calculated band structures (**top row**) and corresponding Seebeck coefficient profiles as a function of chemical potential (**bottom row**) for MPcCl_8_ crystals (M = Co, VO, Zn).

The subsequent step involved computing the electronic band structure, using a 15 × 15 × 15 Monkhorst-Pack *k*-point grid to ensure adequate convergence of the electronic properties. The results indicate that CoPcCl_8_, VOPcCl_8_, and ZnPcCl_8_ are semiconductors with band gaps of 1.187 eV, 0.776 eV, and 1.105 eV, respectively (Figure 10). In all three materials, the Fermi level (*E_F_*) lies closer to the conduction band than to the valence band, suggesting n-type semiconductor behavior. The calculations of the electrical transport coefficients reveal a consistent trend: when the chemical potential (*μ*) is referenced to the *E_F_* and set to zero, the Seebeck coefficient assumes negative values across all studied materials (Figure 10). This unambiguously signifies that electrons are the dominant charge carriers under these conditions.

Previous studies have shown that metal phthalocyanine molecules have two active sites, which means that interaction with analyte molecules can occur through both the central metal atom and the formation of hydrogen bonds between NH_3_ molecules and peripheral macrocycle atoms [2,27,67]. This interaction induces a redistribution of electronic charge within the phthalocyanine framework, altering its electronic structure and, consequently, its electrical conductivity, which is experimentally observed as a change in film resistance. Given that the investigated MPcCl_8_ compounds (M = Co, VO, Zn) exhibit n-type semiconducting behavior, the charge transfer mechanism upon NH_3_ and H_2_S adsorption proceeds as follows: gas molecules, acting as electron donors, transfer a portion of their electron density to the phthalocyanine macrocycles. This electron donation increases the concentration of the majority charge carriers (electrons) in the conduction band, resulting in a measurable decrease in the electrical resistance of the films.

#### 3.2.4. Sensor Characteristics

**Figure 11:**
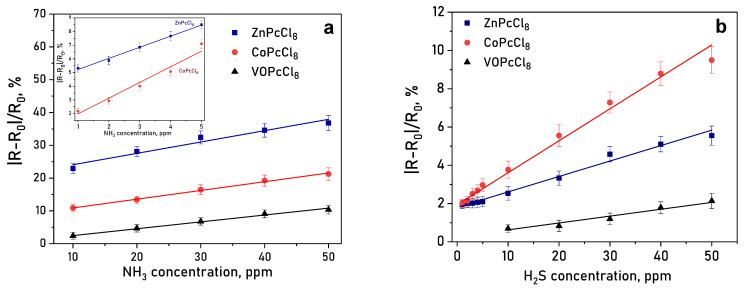
(**a**) Change in the resistance of a CoPcCl_8_ film during the introduction of hydrogen sulfide and subsequent air purging. (**b**) Dependence of the sensor response of MPcCl_8_ films on NH_3_ (**a**) and H_2_S (**b**) concentration (temperature 25 ± 2 °C, RH = 5%).

It is important to mention than the response reversible at room temperature, which is important characteristic of chemiresistive sensors. Response and recovery times are summarized in Table 4. A clear dependence on the central metal ion was observed. ZnPcCl_8_ films exhibited the highest response to NH_3_—over three times greater than CoPcCl_8_ (Figure 11a). CoPcCl_8_ films showed superior sensitivity to H_2_S (Figure 11b). VOPcCl_8_ displayed the weakest response to both analytes.

The distinct chemiresistive behavior of MPcCl_8_ toward NH_3_ and H_2_S can be attributed to the intrinsic electronic and coordination properties of the central metal ions. Cobalt in CoPcCl_8_ adopts a redox-active d^7^ configuration, which allows it to readily engage in charge-transfer interactions with soft Lewis bases. Hydrogen sulfide, with its highly polarizable sulfur atom and available lone pairs, acts as such a ligand and can coordinate to the Co^2+^ center, facilitating partial electron donation into the phthalocyanine *π*-system and thereby enhancing n-type conduction. In contrast, Zn^2+^ in ZnPcCl_8_ possesses a closed-shell d^10^ configuration and is redox-inert under ambient sensing conditions. As a hard Lewis acid, Zn^2+^ preferentially interacts with hard Lewis bases such as ammonia, where the electronegative nitrogen atom provides a localized, high-energy lone pair that forms a relatively stronger donor-acceptor interaction with the Zn center. This interaction effectively modulates the electron population in the conduction band, leading to the pronounced NH_3_ response observed experimentally. In the case of VOPcCl_8_, the presence of the axial V=O bond likely sterically blocks direct access of analyte molecules to the vanadium center, while also withdrawing electron density from the macrocycle, thereby reducing its sensitivity to both gases.

Although this qualitative picture is in good agreement with experimental trends and established principles of coordination chemistry, further modeling of adsorption geometry, binding energies, and charge transfer pathways, as well as experimental measurements such as X-ray and ultraviolet photoelectron spectroscopy (XPS/UPS), would be useful for more thorough verification. Such analyses could directly probe shifts in core-level binding energies (e.g., N 1s, S 2p, or metal 2p/3p levels) and changes in the work function or valence-band edge upon analyte exposure, offering unambiguous evidence of metal-analyte interaction mechanisms. These investigations represent a natural extension of the current work and are planned for future research.

**Figure 12:**
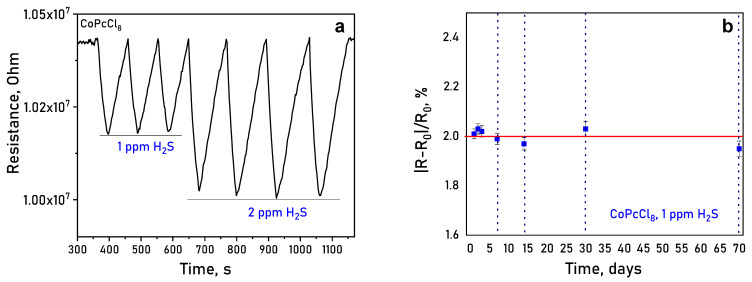
(**a**) Change in the resistance of a CoPcCl_8_ film during repeated exposures of 1 and 2 ppm H_2_S (temperature 25 ± 2 °C, RH = 5%); (**b**) long-term operational results of a CoPcCl_8_ film sensing material to 1 ppm H_2_S.

It was shown using CoPcCl_8_ films as an example that the sensors demonstrated excellent repeatability, with response variations to repeated exposures of 1 and 2 ppm H_2_S remaining within ±5% (Figure 12a). To confirm the long-term stability the response of the same film to 1 ppm of H_2_S was measured after 1, 2, 3, 7, 14, 30 and 70 days (Figure 12b). The change in the sensor response did not exceed the measurement error, which indicated long-term response stability.

**Figure 13:**
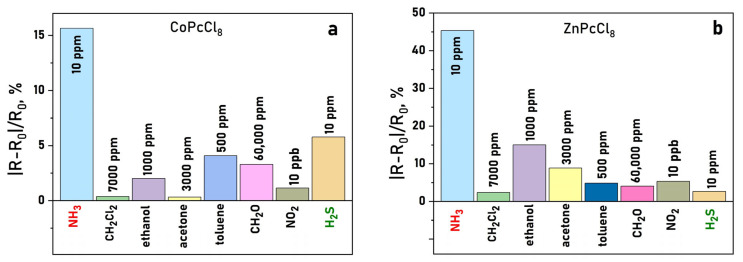
Sensor response of CoPcCl_8_ (**a**) and ZnPcCl_8_ (**b**) NH_3_ (10 ppm), H_2_S (10 ppm), NO_2_ (10 ppm), dichloromethane (7000 ppm), ethanol (1000 ppm), formaldehyde (60,000 ppm) and acetone (3000 ppm) (temperature 25 ± 2 °C, RH = 5%).

Selectivity tests against common interferents, namely acetone, ethanol, toluene, formaldehyde, dichloromethane, and NO_2_, revealed that CoPcCl_8_ and ZnPcCl_8_ films respond preferentially to H_2_S and NH_3_, even when interferents were present at significantly higher concentrations (Figure 13a,b). This highlights their potential for selective detection in complex gas mixtures.

**Figure 14:**
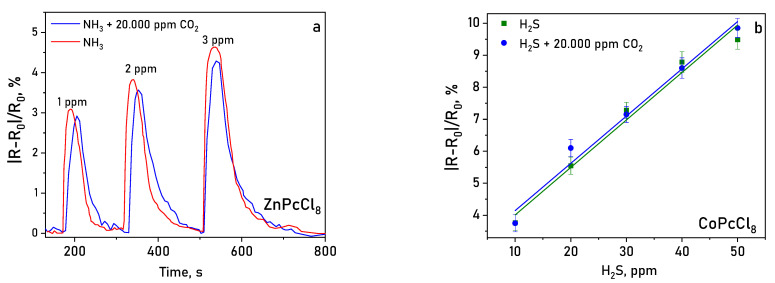
Sensor response of ZnPcCl_8_ (**a**) to NH_3_ (1–3 ppm) and of CoPcCl_8_ (**b**) to H_2_S (10–50 ppm) in the presence of 20.000 ppm CO_2_ (temperature 25 ± 2 °C, RH = 5%).

Layers were tested in a mixture of gases containing 20.000 ppm of CO_2_ (Figure 14). It was shown that the determination of NH_3_ and H_2_S in the presence of carbon dioxide did not lead to a significant change (within the measurement error) in the sensor response measured in air.

**Figure 15:**
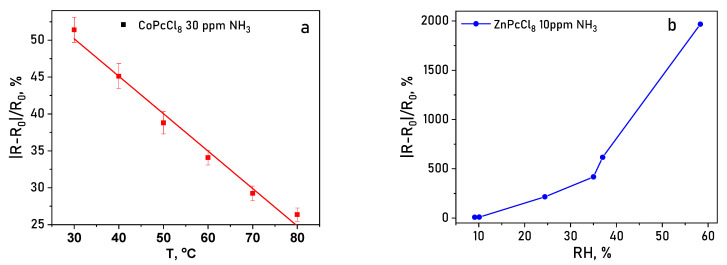
(**a**) Temperature dependence (30–80 °C) of the sensor response of the CoPcCl_8_ toward 30 ppm NH_3_. (**b**) Sensor response of a ZnPcCl_8_ film to 10 ppm NH_3_ measured at various RH.

Operating temperature and relative humidity (RH) strongly modulate sensor performance. Increasing temperature from 30 to 80 °C consistently reduced the response value for both CoPcCl_8_ and ZnPcCl_8_ (Figure 15a), likely due to accelerated desorption of analyte molecules. Conversely, elevated RH dramatically enhanced sensitivity: at 30% RH, the NH_3_ response increased by ~50-fold compared to dry conditions, and at 50% RH, the enhancement reached ~250-fold (Figure 15b). This pronounced humidity dependence suggests that water molecules facilitate analyte adsorption or proton-coupled charge transfer processes at the film surface. Therefore, humidity can significantly affect the sensor’s response, and it must be monitored during the measurement process. To mitigate the effects of humidity, various approaches can be employed. These approaches can be broadly categorized into computational and experimental methods. The computational method involves processing the data obtained from humidity sensors using calibration curves created at different humidity levels. In the experimental approach, waterproof membranes are employed to prevent moisture from reaching the gas-sensitive films.

Table 5 represents the sensor response of halogen-substituted metal phthalocyanine films to 10 ppm ammonia. It is shown that zinc octachlorophthalocyanine (ZnPcCl_8_) films prepared by solution-based methods exhibit a sensor response comparable to, and in some cases superior to, that of other variants. Solution-based film deposition methods offer significant advantages over physical vapor deposition, including lower cost, compatibility with flexible and large-area substrates, and the formation of porous/high-surface-area morphologies that can enhance gas sensitivity. These benefits make our SC film promising for fabricating low-cost phthalocyanine-based sensors with superior response characteristics.

## 4. Conclusions

This study provides a comprehensive investigation of octachlorinated metal phthalocyanines (MPcCl_8_, M = Co, Zn, VO) as active materials for chemiresistive gas sensors. For the first time, the single-crystal structure of CoPcCl_8_ was resolved, confirming a triclinic lattice with cofacial molecular stacks. Comparative structural analysis shows that ZnPcCl_8_ is isostructural with CoPcCl_8_, whereas VOPcCl_8_ crystallizes in a tetragonal system, likely influenced by the axial V=O bond. Thin film morphology and crystallinity are critically dependent on the deposition method: spin-coated films exhibit high crystallinity, and phase similarity to bulk powders, while a PVD method yields poorly ordered or amorphous layers with significantly lower electrical conductivity. Consequently, only SC films demonstrated reliable chemiresistive responses. All MPcCl_8_ SC films displayed n-type semiconducting behavior, as confirmed by both experimental data and DFT-based band structure calculations. Upon exposure to NH_3_ and H_2_S, which are both electron-donating gases, the film resistance decreased reversibly, consistent with increased electron concentration in the conduction band. ZnPcCl_8_ exhibited the highest response to NH_3_ (45.3% at 10 ppm, LOD = 0.8 ppm), whereas CoPcCl_8_ showed superior sensitivity to H_2_S (LOD = 0.3 ppm). As a limitation of these sensors, it should be noted that they are sensitive to humidity, highlighting the importance of environmental conditions in practical deployment: ambient humidity dramatically enhanced sensor response by up to two orders of magnitude. The sensors also demonstrated excellent selectivity against common volatile organic compounds and repeatability over multiple exposure cycles.

## Figures and Tables

**Table 1:** Unit cell parameters and refinement details for CoPcCl_8_.

Compound	CoPcCl_8_
Empirical formula	C_32_H_8_N_8_Cl_8_Co
Formula weight	846.99
Temperature/K	150
Crystal system	triclinic
Space group	P-1
a/Å	3.7062 (10)
b/Å	13.429 (4)
c/Å	14.329 (4)
α/°	83.320 (11)
β/°	89.813 (10)
γ/°	84.227 (11)
Volume/Å^3^	704.7 (4)
Z	1
ρ_calc_g/cm^3^	1.996
μ/mm^−1^	12.145
F (000)	419.0
Crystal size/mm^3^	0.07 × 0.005 × 0.005
Radiation	CuKα (λ = 1.54178)
2Θ range for data collection/°	6.21 to 79.956
Index ranges	−2 ≤ h ≤ 3, −11 ≤ k ≤ 10, −11 ≤ l ≤ 11
Reflections collected	2386
Independent reflections	840
R_int_	0.1303
Data/restraints/parameters	840/120/199
Goodness-of-fit on F^2^	0.977
Final R indexes [I ≥ 2σ (I)]	R_1_ = 0.0573, wR_2_ = 0.1092
Final R indexes [all data]	R_1_ = 0.1429, wR_2_ = 0.1415
Largest diff. peak/hole/e Å^−3^	0.36/−0.49
CCDC No	2503921

**Table 2:** Unit cell parameters for ZnPcCl_8_ and VOPcCl_8_.

Compound	ZnPcCl_8_	VOPcCl_8_
Formula	C_32_H_8_Cl_8_N_8_Zn	C_32_H_8_Cl_8_N_8_VO
Formula weight	853.49	855.04
Crystal system	triclinic	tetragonal
Space group	P-1	I4/m
a/Å	3.82 (4)	19.866 (6)
b/Å	13.54 (12)	19.866 (6)
c/Å	14.24 (10)	3.793 (1)
α/°	83.44 (1)	90
β/°	90.14 (2)	90
γ/°	85.86 (2)	90
Oбъeм/Å^3^	730.8	1496.9
Z	1	2
ρ_calc_g/cm^3^	1.942	1.897

**Table 3:** Assignments of the most intense bands in IR spectra of MPcCl_8_.

Experimental Wavenumbers, cm^−1^			Assignment
CoPcCl_8_	ZnPcCl_8_	VOPcCl_8_	
431	-	428	{C_γ_, H} OOP * vibrations
440	434	436	N_α_-M-N_α_, C_β_-C_γ_-C_δ_, C_γ_-C_δ_-Cl, {C_γ_, H} OOP vibrations
505	496	505	N_α_-M, C_γ_-Cl, C_β_-C_γ_-C_δ_
552	544	546	N_α_-M-N_α_, C_α_-N_α_-C_α_, C_δ_-C_δ_-Cl, C_α_-N_β_-C_α_
662	662	662	C_γ_-Cl, C_β_-C_β_-C_γ_, C_β_-C_γ_-C_δ_
708	702	706	N_α_-M, N_α_-C_α_-C_β_, C_α_-C_β_-C_β_, C_δ_-Cl
750	743	748	{C_α_-N_α_-C_α_-N_β_} zig-zag OOP vibration, H OOP motions
763	-	766	{C_α_-N_α_-C_α_-N_β_} zig-zag OOP vibration, H OOP motions
785	781	781	M-N_α_, M-N_α_-C_α_, C_α_-N_β_-C_α_, C_α_-C_β_-C_γ_, C_β_-C_γ_-H, C_δ_-Cl
851	839	849	M-N_α_, C_α_-N_β_-C_α_, C_γ_-C_δ_-C_δ_, C_β_-C_γ_-H, C_δ_-Cl
889	891	895	{C_γ_, H} OOP vibrations
966	945	955	C_β_-C_γ_-H, C_δ_-C_δ_-Cl, C_γ_-C_δ_-C_δ_, C_δ_-Cl
-	-	1012	V=O
1074	1069	1072	C_β_-C_γ_-H, C_δ_-Cl, C_α_-C_β_-C_γ_
1089	1088	1088	C_α_-N_α_-C_α_, C_β_-C_γ_-H, C_δ_-Cl, C_α_-N_α_
1140	1132	1132	C_α_-N_α_-C_α_, C_β_-C_γ_-H, C_δ_-Cl, C_α_-N_α_, benzene breathing
1202	1190	1190	C_α_-C_β_-C_γ_, C_β_-C_γ_-H, C_α_-N_α_-C_α_, C_α_-N_α_
1292	1288	1290	C_α_-N_α_-C_α_, C_α_-C_β_-C_β_, C_δ_-C_δ_, C_α_-C_β_, C_δ_-C_γ_-H
1338	1329	1329	C_β_-C_β_, C_δ_-C_δ_, C_γ_-C_δ_, C_α_-N_α_-C_α_
1389	1381	1381	C_γ_-C_δ_, C_β_-C_γ_-H, C_γ_-C_δ_-C_δ_, N_α_-C_α_-C_β_
1418	1411	1416	C_γ_-C_δ_, C_β_-C_γ_-H, C_δ_-C_δ_-Cl, C_α_-C_β_
1458	1456	1454	N_α_-C_α_-N_β_, C_α_-N_β_, C_α_-C_β_, C_β_-C_β_, C_β_-C_γ_-H
1524	1485	1489	C_α_-N_β_, C_α_-N_β_, C_α_-C_β_, C_β_-C_γ_-H
1582	1568	1570	C_β_-C_β_, C_δ_-C_δ_
1605	1599	1607	C_α_-C_β,_ C_β_-C_γ_, C_γ_-C_δ_

* OOP—out-of-plane vibrations.

**Table 4:** Limit of detection, response and recovery (10 ppm) times for SC MPcCl_8_.

MPcCl_8_	LOD, ppm		Response Time, s		Recovery Time, s	
	NH_3_	H_2_S	NH_3_	H_2_S	NH_3_	H_2_S
CoPcCl_8_	1	0.3	30	35	180	140
ZnPcCl_8_	0.8	1	45	15	150	80
VOPcCl_8_	3.5	5	35	20	65	100

**Table 5:** Sensor response of the films of halogenated metal phthalocyanines to ammonia (10 ppm).

Phthalocyanine Molecule	Deposition Technique	Response, % 10 ppm NH_3_	Reference
ZnPcF_4_-p	PVD	16	[2]
ZnPcCl_4_-p	PVD	27	[2]
CoPcF_4_-p	PVD	79	[67]
VOPcCl_4_-p	PVD	26.4	[27]
CoPcCl_4_-p	SC	9.3	[60]
CoPcCl_8_	SC	15.6	This work
ZnPcCl_8_	SC	45.3	This work

## Data Availability

The original contributions presented in this study are included in the article. Further inquiries can be directed to the corresponding author.
